# Low-Vacuum Catalyst-Free Physical Vapor Deposition and Magnetotransport Properties of Ultrathin Bi_2_Se_3_ Nanoribbons

**DOI:** 10.3390/nano13172484

**Published:** 2023-09-03

**Authors:** Raitis Sondors, Kiryl Niherysh, Jana Andzane, Xavier Palermo, Thilo Bauch, Floriana Lombardi, Donats Erts

**Affiliations:** 1 Institute of Chemical Physics, University of Latvia, LV-1586 Riga, Latvia; 2 Quantum Device Physics Laboratory, Department of Microtechnology and Nanoscience, Chalmers University of Technology, 41296 Gothenburg, Sweden; 3 Faculty of Chemistry, University of Latvia, LV-1586 Riga, Latvia

**Keywords:** bismuth selenide, ultrathin nanoribbons, bulk-free topological insulator, catalyst-free physical vapor deposition, magnetotransport properties

## Abstract

In this work, a simple catalyst-free physical vapor deposition method is optimized by adjusting source material pressure and evaporation time for the reliable obtaining of freestanding nanoribbons with thicknesses below 15 nm. The optimum synthesis temperature, time and pressure were determined for an increased yield of ultrathin Bi_2_Se_3_ nanoribbons with thicknesses of 8–15 nm. Physical and electrical characterization of the synthesized Bi_2_Se_3_ nanoribbons with thicknesses below 15 nm revealed no degradation of properties of the nanoribbons, as well as the absence of the contribution of trivial bulk charge carriers to the total conductance of the nanoribbons.

## 1. Introduction

Bismuth selenide (Bi_2_Se_3_) is a semiconducting material that belongs to the 3D topological insulators (3D TIs) [1,2,3]. The properties of Bi_2_Se_3_ nanostructures have been extensively studied for potential applications in a variety of fields, including spintronic devices [4,5], sensors [6,7,8], photodetectors [9,10,11], battery electrodes [12,13,14], as well as thermoelectric [15,16,17,18,19] and nanoelectromechanical devices [16]. While unique surface states have made Bi_2_Se_3_ an attractive candidate for the realization of various quantum phenomena, they are often masked by the contribution of bulk charge carriers [20]. It has been reported that the bulk conductivity of TIs was successfully suppressed by introducing chemical compensating dopants into nanostructures [21,22,23]. However, due to scattering on dopant impurities in such doped materials, the mobility of charge carriers often does not exceed ~500 cm^2^·(Vs)^−1^ [23,24]. Thus, undoped Bi_2_Se_3_ nanostructures as ultrathin films, nanoribbons (elongated nanostructures with a rectangular cross-section in which the width is much larger than the thickness) [3,25] and nanoplates are great candidates for the effective utilization of their topological surface states due to the high surface-to-volume ratio, suppressing the bulk charge carriers [26] and high mobility.

On the other hand, the use of TIs nanoplates, nanowires, and nanoribbons in various types of electrical applications requires the transfer of the nanostructures to a dielectric substrate. While applying an electrostatic field via gate electrode to the TI-based device, the additional carriers dope the nanostructure, forming an accumulation layer at the dielectric–topological insulator interface. As a result, the energy band bending effect occurs [22]. The depth of the accumulation layer D can be estimated by solving Poisson equation in the full depletion approximation to yield D = (2κε_0_ΔE/(e^2^n))^−1/2^ [22,24], where κ is the dielectric permittivity, ΔE is the shift of Fermi energy across the bang-bending region due to the applied gate voltage (ΔE≤100–300 meV (TIs bandgap [27])), and n is the carrier density. The values of the accumulation layer depth ranging from 10 to 35 nm for samples with carrier density n ~10^19^ cm^−3^ were reported in the literature [22,23,24,28,29,30,31,32]. To fully suppress bulk conduction, the sample thickness should be smaller than D so that the gate can effectively modulate the charge carrier density of the entire sample. Considering the slight variation of carrier density between the nanoribbons, as well as the non-ideality in the samples, such as compositional inhomogeneity and the possible presence of the defect states, the thickness of 15 nm can be chosen as a top limit reference for optimizing growth parameters in order to achieve an increased yield of such nanoribbons. In addition, some unique effects, such as the formation of a hybridization gap, resulting in the disengaging of electrical and thermal conductance, may occur in topological insulators with thickness reduced below 10 nm [33].

The physical vapor deposition (PVD) method is attractive for the synthesis of Bi_2_Se_3_ nanoribbons due to its simplicity and easily adjustable synthesis parameters such as partial pressure of vaporized source material, heating and deposition time and rate, substrate temperature, which make it an ideal candidate for high-yield synthesis of chemically pure nanostructures. Another advantage of the catalyst-free PVD is no need for expensive single-crystalline substrates. Previously, the successful synthesis of Bi_2_Se_3_ nanoribbons has been demonstrated via catalyst-free [34,35] and catalyst-assisted [36,37,38] PVD techniques. Both catalyst-free and catalyst-assisted methods allowed for freestanding Bi_2_Se_3_ nanoribbons to be obtained, permitting their easy handling and transfer to the desired substrates and positions [26,34,36]. However, the thicknesses of the vast majority of these nanoribbons ranged from ~20 to ~100 nm, with a negligible number of nanoribbons with thicknesses below 15 nm [39]. The nanoribbon thicknesses above 20 nm are too large for utilizing TI properties of the nanoribbons as the increased thickness increases the contribution of bulk conductivity. In turn, the use of the ultrathin nanoribbons obtained in these syntheses is complicated by their extremely small number. Previously, our group reported strong gate tunability of the Fermi level (ambipolar transport) for a 9 nm-thick Bi_2_Se_3_ nanoribbon occasionally formed during the catalyst-free physical vapor deposition [38]. These findings became a trigger for the optimization of the synthesis process to achieve an increased yield of ultrathin nanoribbons.

In the present work, the main parameters (synthesis temperature, time, and vapor pressure) of previously developed catalyst-free physical vapor deposition method for the obtaining of freestanding Bi_2_Se_3_ nanoribbons [34] were systematically optimized to achieve the outcome of the Bi_2_Se_3_ nanoribbons with thicknesses below 15 nm. The optimum parameters were determined for maximizing the yield of ultrathin nanoribbons. To reveal their topological states, magnetotransport properties were investigated for the ultrathin (≤15 nm) Bi_2_Se_3_ nanoribbons obtained using the optimized synthesis parameters.

## 2. Materials and Methods

### 2.1. Synthesis of Bi_2_Se_3_ Nanoribbons

Bi_2_Se_3_ nanoribbons were synthesized via catalyst-free physical vapor deposition, similarly as reported in [34] in a GSL-1100X tube furnace (length 60 cm, diameter 46 mm) (MTI Corporation, Richmond, CA, USA). The calibration curve for the temperature profile along the furnace tube was taken prior to the synthesis of the Bi_2_Se_3_ nanostructures under the same conditions (temperature, time, and pressure) as for the synthesis of the nanostructures using a K-type thermocouple installed in the flange of the furnace tube and allowing to monitor the temperature in different locations inside it. The pressure inside the furnace tube was measured using NIST Traceable Digital Convection Vacuum Gauge (Stinger, InstruTech, Inc., Longmont, CO, USA), which passed the certified calibration for direct readout of nitrogen. The maximum expected error for the pressure readout does not exceed 0.1 Torr). Bi_2_Se_3_ flakes (99.999%, Sigma Aldrich, St. Louis, MO, USA) were used as the source material, and 25 × 75 mm glass microscope slides were used as the substrate. 90 mg of the source material was placed in the center of the tube furnace, where the temperature during the synthesis reached 585 °C. The substrate was placed downstream from the source material in a zone where the temperature (based on the calibration curve) during the synthesis reaches 320 °C at the cold side of the substrate to 450 450 °C at the hot side of the substrate. The temperature in the center of the substrate during the synthesis was 400–410 °C. The furnace tube was first flushed with N_2_ gas for 5 min to create an inert atmosphere. The initial temperature in all syntheses was 25 °C. The furnace heating rate from the room temperature to 575–590 °C was ~12.5 °C/min (45 min). The start pressure in the furnace tube varied from 0.1 to 18 Torr. During the deposition process, the furnace was kept at a temperature of 575–590 °C for a time ranging from 0 up to 30 min, after which the furnace heater was turned off to cool down naturally. After the temperature in the furnace center had decreased to 540 °C, an N_2_ gas flow was introduced with a dynamic pressure of ~25 Torr. After the temperature had decreased to 475 °C, the flow was terminated, and the tube was rapidly filled with N_2_ to an atmospheric pressure.

### 2.2. Characterization of Morphology of Bi_2_Se_3_ Nanoribbons

Field emission scanning electron microscope (SEM, Hitachi S-4800, Hitachi Ltd., Chiyoda, Tokyo, Japan) equipped with an energy-dispersive X-ray (EDX) analyzer Bruker XFLASH 5010 (Bruker Corporation, Billerica, MA, USA) was used for the inspection of morphology and chemical composition of nanostructures grown on the substrate. The as-grown Bi_2_Se_3_ nanoribbons were mechanically transferred from the glass substrate to flat Si substrates, and an atomic force microscope (AFM, Bruker Dimension ICON, Bruker Corporation, Billerica, MA, USA) was used to measure the thicknesses of more than 1000 individual Bi_2_Se_3_ nanoribbons. The *R* language for statistical computing was used for data analysis and visualization [40].

### 2.3. Magnetotransport Measurements

Electron beam lithography (JEOL JBX 9300FS, JEOL Ltd., Akishima, Tokyo, Japan), Ar-ion beam etching (Oxford Ionfab 300 Plus, Oxford Instruments, Abington, UK) and vacuum evaporator (Lesker PVD 225, Kurt J. Lesker Company, Pittsburgh, PA, USA) were used to create electrical contacts to individual nanoribbons. Magnetotransport measurements were performed in the four-terminal configuration using the physical property measurement system (Quantum Design DynaCool (14T), Quantum Design, Inc., San Diego, CA, USA) in the temperature range 2–300 K.

## 3. Results and Discussion

### 3.1. Optimization of Synthesis Parameters for Obtaining Ultrathin Bi_2_Se_3_ Nanoribbons

**Figure 1:**
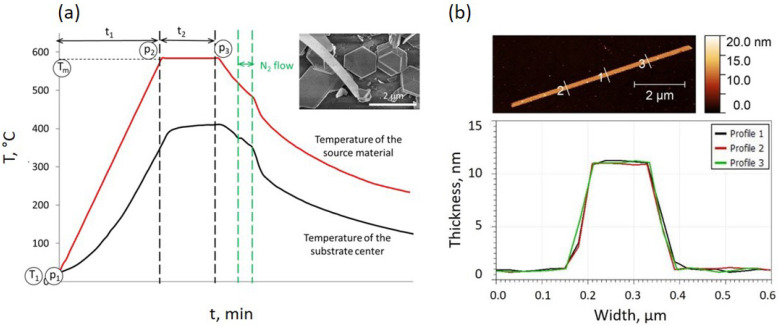
(**a**) An example of temperature profiles measured in real-time prior to the synthesis of Bi_2_Se_3_ nanostructures under the same conditions (temperature, time, and pressure) as for the synthesis of the nanostructures: at the center of the furnace (location of the Bi_2_Se_3_ source material—solid red line) and at the center of the substrate (the region where the stoichiometric Bi_2_Se_3_ nanoribbons are obtained—solid black line); Inset—secondary electron SEM image of a Bi_2_Se_3_ nanoribbon growing from the edge of Bi_2_Se_3_ nanoplate-seed; (**b**) atomic force microscope image of ~10 nm thin Bi_2_Se_3_ nanoribbon (top) and height profiles at three different positions demonstrating an identical shape and thickness of the cross-section of the nanoribbon (bottom).

As reported previously [34], the growth of the Bi_2_Se_3_ nanoribbons during the catalyst-free vapor-solid deposition is initiated by the temporary N_2_ gas flow, which is introduced in the furnace tube during the cooling stage. The real-time temperature profiles in the center of the furnace tube and in the center of the substrate during the synthesis and at the moment of N_2_ flow introduction are shown in Figure 1a. Later, it was demonstrated in [41] that the growth of the Bi_2_Se_3_ nanoribbons in the catalyst-free PVD process occurs from the edges of the Bi_2_Se_3_ nanoplates seeds (Figure 1a, inset). The stoichiometry of the obtained nanoribbons was confirmed by the EDX analysis, showing a Bi:Se proportion of 37 ± 3 (Bi):62 ± 4 (Se). The representative AFM image and related height profiles illustrate the rectangular cross-section of the nanoribbon with the width exceeding the thickness of the nanoribbon by a factor of ~13 (Figure 1b). Height profiles obtained at three different positions across the nanoribbon match well with each other, indicating uniform shape and thickness of the nanoribbon’s cross-section.

**Figure 2:**
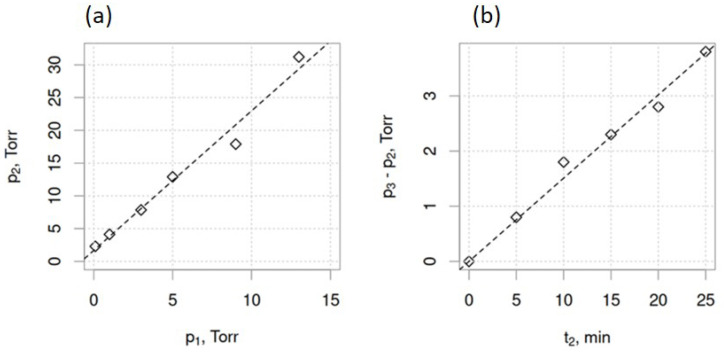
Charts illustrating relation between the (**a**) pressure p_2_ at the end of the furnace heating to maximal synthesis temperature and the initial pressure p_1_, and (**b**) between the heating time t_2_ of the source material at maximal temperature T_m_ and the pressure increase during the t_2_ time. All p_1_, p_2_, and p_3_ measurement errors did not exceed 1% of the measured value.

The growth of the Bi_2_Se_3_ nanoribbons from the edges of the nanoplates can be explained by the Bi_2_Se_3_ deposition kinetics. As Bi_2_Se_3_ is a highly anisotropic layered material, its growth occurs much faster in the lateral direction (perpendicular to the crystallographic *c*-axis) compared to the vertical. As the top and bottom surfaces of the Bi_2_Se_3_ nanoplates are chemically saturated with selenium [42], the Bi and Se adatoms cannot form covalent bonds with the atoms on the surface and diffuse to the edges of the growing nanoplate, having a number of dangling bonds. Without the carrier gas flow, the growth of the Bi_2_Se_3_ nanoplates is a result of a natural diffusion of the vaporized source material to the substrate and following the motion of the adatoms to the energetically favorable edges of the nanoplates. This leads to the formation of symmetrical nanoplates or stacks of nanoplates (Figure 1a, inset). However, the introduction of the N_2_ gas flow provides a rapid increase in the concentration of the evaporated source material near the substrate, accompanied by the change of the temperature gradient along the substrate due to the approaching hot carrier gas from the center of the furnace tube (Figure 1a, black solid line) as well as an insignificant change of the temperature of the source material (Figure 1a, red solid line). The introduction of the gas flow results in directed diffusion of the adatoms, leading to a much faster growth rate in the N_2_ flow direction and to the formation of long crystalline nanoribbons starting from the edge of the nanoplates [41]. The rapid decrease in the temperature after the N_2_ gas flow is turned off (Figure 1a) is related to the filling of the furnace tube with N_2_ up to atmospheric pressure for the termination of the nanoribbon growth process. The thickness of the nanoribbons is most likely determined by the thickness of the nanoplates or, in the case of a step-like structure of the nanoplates, by the thickness of the step. In turn, the growth rate and, consequently, the thickness of the nanoplates/nanoplate steps depend on the amount and diffusion rate of the vaporized source material. The rate of evaporation can be approximated by the Hertz–Knudsen evaporation equation [43]:

$$\;\frac{\mathrm{dN}}{\mathrm{dt}}=\frac{\alpha_{e}N_{A}\left(p_{e}-p_{h}\right)}{\sqrt{2\pi \mathrm{MRT}}},$$

where N—number of evaporated atoms per surface area, t—time, α_e_—the coefficient of evaporation, N_A_—Avogadro number, p_e_—equilibrium pressure, p_h_—hydrostatic pressure, M—molecular weight of the evaporated species, R—is universal gas constant, T—absolute temperature. Considering the constant initial mass of the source material and its heating rate in all syntheses, the amount and diffusion rate of the vaporized source Bi_2_Se_3_ is governed by the three variable parameters of the synthesis: maximal heating temperature of the source material T_m_ (Figure 1a), the initial pressure in the furnace tube p_1_ (Figure 1a), which determines the pressure p_2_ at the end of the heating stage (Figure 2a), and time t_2_, during which the source material is kept at T_m_ (Figure 1a). In the synthesis process, t_2_ determines the pressure p_3_ at the end of the heating stage (Figure 1a and Figure 2b).

In the previously demonstrated syntheses of the Bi_2_Se_3_ nanoribbons on glass [34,39] and anodized alumina [41] substrates, these parameters were T_m_ = 585 °C, p_1_ = 0.5–5 Torr and t_2_ = 15 min. The syntheses based on these parameters resulted in the formation of freestanding nanoribbons with thicknesses starting from 7–9 nm [39] and up to 100 nm [34,39,41] within one batch of nanoribbons. However, the vast majority of the Bi_2_Se_3_ nanoribbons had thicknesses between 25 nm [26] and 80 nm [41], with the number of ultrathin nanoribbons being negligible, which makes their use for practical applications extremely challenging.

To optimize the synthesis parameters to reach a sufficient outcome of the Bi_2_Se_3_ nanoribbons with reduced thicknesses, the parameters T_m_, p_1_ and t_2_ were varied in the ranges 575–590 °C, 0.1–18 Torr and 0–30 min, respectively, to determine the optimal growth conditions for the nanoribbons with thicknesses below 25 nm, and especially for obtaining ultrathin nanoribbons with thicknesses below 10–15 nm. The tested synthesis parameters and the outcome of nanoribbons of different thicknesses are summarized in Table 1, with emphasis on the proportions of nanoribbons with thicknesses in the ranges of <10 nm, 10–15 nm, 15–20 nm, 20–25 nm, and >25 nm.

Variation of T_m_ did not result in the increased outcome of the ultrathin nanoribbons compared to the standard T_m_ = 585 °C (yellow part of Table 1). Reduction of T_m_ by 10 °C (from 585 °C down to 575 °C) resulted in the outcome of nanoribbons with thicknesses above 25 nm, with the mean value of the nanoribbon thickness of 52 nm (Table 1, row 1), which is higher in comparison with the value of 36 nm obtained for the similar synthesis performed with the T_m_ = 585 °C (Table 1, row 6). In turn, an increase in T_m_ by 5 °C up to 590 °C resulted in the poor quality of the nanoribbons, expressed in a high number of defects in their crystal structure, presumably due to the excess of Bi (Table 1, row 2), which was confirmed by the EDX analysis which showed Bi:Se proportion of (45 ± 3)% (Bi):(55 ± 3)% (Se). Thus, T_m_ = 585 °C, which was used in the previously developed synthesis, and resulted in a reliable outcome of stoichiometric nanoribbons, was selected as the constant parameter. Furthermore, only parameters t_2_ (green part of Table 1) and p_1_ (blue part of Table 1) were varied.

**Figure 3:**
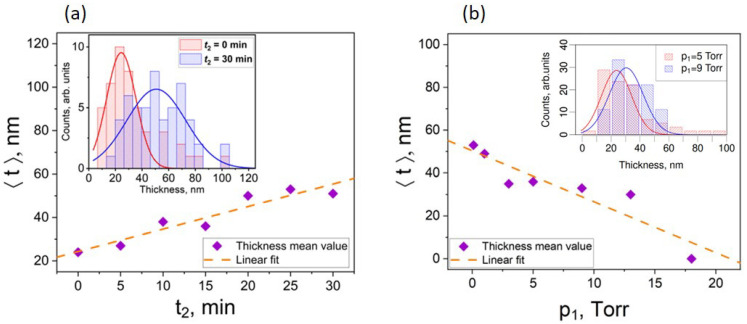
Bi_2_Se_3_ nanoribbon mean thickness ⟨t⟩ vs. (**a**) time t_2_ at a constant initial pressure p_1_ = 5 Torr, and (**b**) vs. the initial pressure p_1_ at constant time t_2_ = 0 min. The orange dashed lines correspond to a linear fit of the experimental data. Insets: Bi_2_Se_3_ nanoribbon thickness histograms for t_2_ = 0 min and 30 min at constant p_1_ = 5 Torr (left), and for p_1_ = 5 Torr and 9 Torr at constant t_2_ = 0 min (right). Solid lines correspond to the Gaussian distribution of experimental data, from which the mean thickness ⟨t⟩ was calculated.

Generally, the mean thickness of the nanoribbons linearly decreased with the decrease in the source material heating time t_2_ while keeping the pressure p_1_ constant (Figure 3a, and the green part of Table 1, including row 9), which is most likely related to the decreased amount of the evaporated source material, and consequently, the thickness of the nanoplate seeds. In contrast, an increase in the initial pressure p_1_ while keeping t_2_ constant resulted in a slight decrease in the mean thickness ⟨t⟩ of the nanoribbons from ~50 nm down to 0 nm when the p_1_ increased from 0.1 to 18 Torr (Figure 3, blue part of Table 1, including row 9).

As can be seen from the inset in Figure 3a, an increase in the t_2_ at constant pressure p_1_ impacts not only the mean thickness of the nanoribbons ⟨t⟩, but also the distribution of the actual thicknesses of the nanoribbons. The thickness distribution at t_2_ = 0 min has a majority of the nanoribbons within the range 10–40 nm with the maximum at the nanoribbon thickness values of ~20 nm (Figure 3a (inset), red pillars). In contrast, the thickness distribution of the nanoribbons obtained in the synthesis with t_2_ = 30 min is more uniform and has maximum shifted to the thicknesses 50 nm (Figure 3a (inset), blue pillars). Consequently, t_2_ = 0 min is an optimal choice for obtaining the highest number of ultrathin nanoribbons. The thickness distribution for most of the nanoribbons obtained in the synthesis with p_1_ = 5 Torr is mainly between 0 and 40 nm, with the maximum at the nanoribbon thickness 10–20 nm (Figure 3b (inset), red pillars). In contrast, the thickness distribution for the nanoribbons obtained in the synthesis with p_1_ = 9 Torr was wider and shifted to the higher thickness values, covering the range 10–60 nm (Figure 3b (inset), blue pillars), with the maximum shifted to the 20–30 nm-thick nanoribbons, which may be related to the formation of thicker nanoplates seeds as explained further in the text. Thus, the p_1_ of 5 Torr may be considered as optimal pressure for the obtaining of ultrathin nanoribbons.

**Figure 4:**
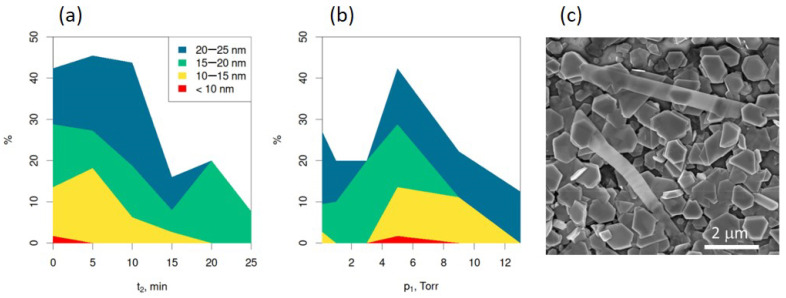
Stacked area plots of Bi_2_Se_3_ nanoribbon mean thicknesses (**a**) from syntheses with p_1_ = 5.0 Torr with various heating times t_2_, and (**b**) t_2_ = 0 min with various starting pressure p_1_. (**c**) The secondary electron scanning electron microscope image of ultrathin Bi_2_Se_3_ nanoribbons grown from the step-like structure at the top surfaces of Bi_2_Se_3_ nanoplate seeds.

The ranges of the synthesis parameters t_2_ and p_1_ suitable for obtaining ultrathin Bi_2_Se_3_ nanoribbons of thicknesses <10 nm, 10–15 nm, 15–20 nm, and 20–25 nm are illustrated in Figure 4a and b, respectively. While nanoribbons with thicknesses 20–25 nm may be obtained within the wide range of parameters with t_2_ ranging from 0 to 25 min and p_1_ ranging from 0.1 to 13 Torr (Figure 4a,b, blue area), the optimal parameters, resulting in a yield of ~42–46% of nanoribbons with these thicknesses are T_m_ = 585 °C, t_2_ = 0–10 min, and p_1_ = 5 Torr (Table 1, rows 7–9). These synthesis parameters also result in narrow nanoribbon thickness distributions, with most of the nanoribbons having thicknesses below 25 nm. As suggested by the Hertz–Knudsen evaporation equation, increasing the initial pressure effectively reduces the diffusion rate of the source material, resulting in slower nucleation and growth of the nanoplate seeds; however, this does not hamper their formation.

However, increasing the time t_2_ above 10 min while keeping p_1_ = 5 Torr results in an increase in the yield of Bi_2_Se_3_ nanoribbons with thicknesses above 25 nm up to ~60–89% (Table 1, rows 3–6), which indicates that the growth of nanoplates seeds starts before the heating stage at constant T_m_ (Figure 1) and continues during the full period of the heating time t_2_. A similar increase in the outcome of the nanoribbons with thicknesses above 25 nm was observed with the decrease in p_1_ below 5 Torr (Table 1, rows 10–12) or its increase above 5 Torr (Table 1, rows 13–14) while keeping t_2_ = 0 min. The decrease in p_1_ may lead to the formation of thicker nanoplate seeds due to the increased evaporation and diffusion rate of the source material. In turn, the increase in p_1_ may result in the formation of nanoplates with a high number of surface defects, hampering the diffusion of adatoms to the edges of the nanoplates and promoting the formation of the step-like structure of the nanoplates with high step thicknesses. The evaporation equation suggests that no more evaporation occurs when the hydrostatic pressure exceeds the equilibrium pressure of the source material. This was experimentally confirmed by observing that synthesis at a pressure p_1_ = 18 Torr did not yield any nanoribbons (Table 1, row 15), indicating that the synthesis pressure was close to or above the equilibrium pressure of the source material.

While the nanoribbons with thicknesses of 15–20 nm may also be obtained in quite wide range of synthesis parameters: t_2_ ranging from 0 to 25 min and p_1_ ranging from 0.1 to 13 Torr (Figure 4a,b, green area), the range of synthesis parameters for obtaining nanoribbons with thicknesses 10–15 nm required reduction of t_2_ below 20 min and p_1_ falling in the range 3–13 Torr (Figure 4a,b, yellow area). For both 10–15 nm and 15–20 nm thickness ranges, the initial pressure p_1_ = 5 Torr was found to be optimal for obtaining the highest yield of the nanoribbons. However, for obtaining maximal yield (~27–29% of 15–20 nm thin nanoribbons and ~13.5–18% of 10–15 nm thin nanoribbons, Table 1, rows 8. and 9), the time t_2_ had to be reduced to 0–5 min with the optimal value of t_2_ = 0 min. While there was no significant difference in the yields of the 15–20 nm thin nanoribbons obtained in the syntheses with t_2_ being 0 min and 5 min (28.8% vs. 27.3%), the difference in the yields of the 10–15 nm at these values of t_2_ was noticeable (13.6% vs. 18.2%). Presumably, this effect may be related to the dynamics of the formation of the step-like structure of the nanoplates seeds formed before the start of the second stage of the synthesis—heating the source material at constant temperature T_m_—and further initiation of the nanoribbon growth from the edges of these steps. In the synthesis with t_2_ = 0, the step-like morphology starts its formation, and at t_2_ = 5 min, more step-like structures are formed, resulting in an increased yield of the nanoribbons. This hypothesis is indirectly supported by the fact that the nanoribbons with thicknesses below 10 nm (Figure 4a,b, red area) can be formed in the narrow range of the synthesis parameters of t_2_ varying from 0 to 5 min and p_1_ varying between 3 and 9 Torr with the optimal parameters p_1_ = 5 Torr and t_2_ = 0 min when the yield of 1.7% of the total number of characterized nanoribbons can be reached (Table 1, row 9). These nanoribbons were grown from the edges of newly formed step-like structures on the surfaces of the Bi_2_Se_3_ nanoplates seeds, as illustrated in Figure 4c. It should be noted that the minimal detected thickness of the nanoribbons obtained under these synthesis parameters was 8 nm, which presumably may be the lower limit for the controlled synthesis of ultrathin nanoribbons by physical vapor deposition.

### 3.2. Magnetotransport Properties of Ultrathin Bi_2_Se_3_ Nanoribbons

**Figure 5:**
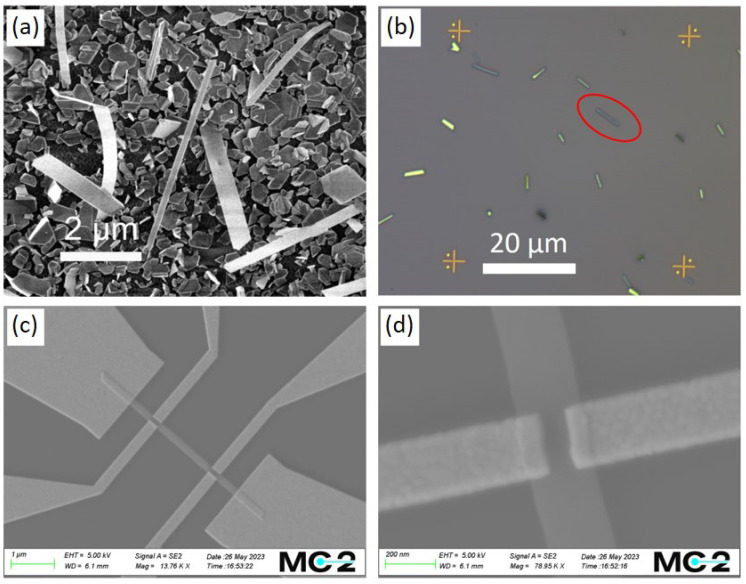
(**a**) Secondary electron SEM image of grown freestanding Bi_2_Se_3_ nanoribbons on a glass substrate. (**b**) Optical image of transferred nanoribbons to Si/SiO_2_ substrate. The red circle highlights the nanoribbon (15 nm thick) selected for further fabrication. (**c**) SEM image of Bi_2_Se_3_ nanoribbon-based (from (**b**)) device with patterned electrodes for the Hall-effect measurement; (**d**) Hall bars overlapping the Bi_2_Se_3_ nanoribbon; this configuration requires a correction factor g when calculating the n_3D-Hall_ concentration.

The freestanding stoichiometric Bi_2_Se_3_ nanoribbons synthesized with adjusted parameters on a glass substrate (Figure 5a) were transferred to Si/SiO_2_ (7 × 7 mm) substrates with marks via simple flip-chip (slight pressing of the chip to the glass with nanoribbons) method. The transferred nanoribbons were analyzed using an optical (Figure 5b) and atomic force microscope (Figure 1b) to distinguish the thinnest ones. The selected ultrathin nanoribbons were patterned using electron-beam lithography to create electrical contacts in a four-terminal measuring configuration. Since the surfaces of Bi_2_Se_3_ nanoribbons oxidize in the air faster than a cleaved single crystal [44], and for our nanoribbons, synthesized by the standard method, ~1 nm oxide layer formed in one week, but after 3 years, their surface is covered with a 10 nm-thick oxide layer [45] (other groups also show higher oxidation rates ~2 nm oxide layer is formed in 2 days of exposure to air [46]), the samples were stored in an inert atmosphere after the synthesis to reduce the thickness of the formed native oxide layer. To remove the oxide layer and provide an ohmic contact, approximately 3–4 nm thin layers were etched off the nanoribbon surfaces with Ar ions prior to the formation of the metal electrodes to the nanoribbons by the evaporation method. After etching, the sample was immediately loaded into a vacuum chamber of a metal evaporator. To enhance adhesion, an extremely thin adhesion layer of Ti (3 nm) was deposited before the gold evaporation (80 nm). SEM image of a device with Hall bar electrodes geometry based on a single ultrathin (15 nm) nanoribbon is shown in Figure 5c. The six-contacts geometry (two large electrodes at the ends of nanoribbon for passing current and two pairs of Hall bars) allows measurements of the longitudinal (V_xx_) and transverse (V_xy_) voltages in a four-probe configuration, thus eliminating the contact resistance effects. However, the overlapping of the nanoribbon with gold electrodes (Figure 5d) results in an error in Hall voltage measurements due to the non-ideal Hall bar geometry. Thus, similarly to reported in [39], the measured value of V_xy_ must be corrected using a numerically calculated geometrical correction factor for the specific device geometry.

**Figure 6:**
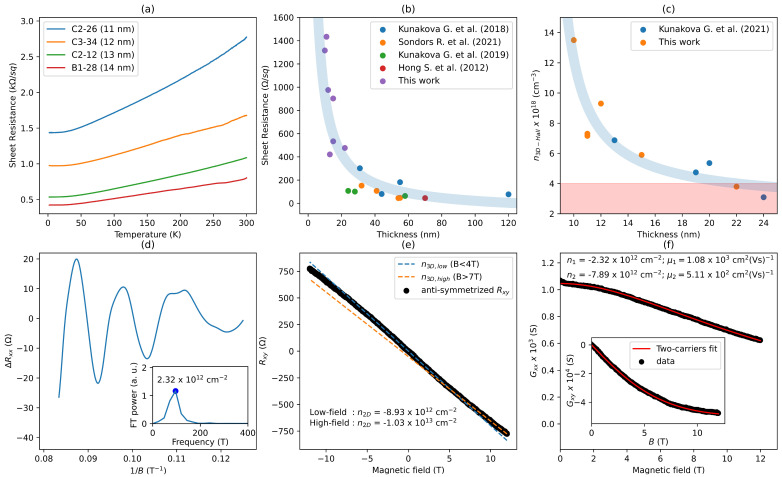
Magnetotransport of Bi_2_Se_3_ nanoribbons with thickness below 15 nm. (**a**) Sheet resistance as a function of temperature; (**b**) Sheet resistance as a function of nanoribbon thickness measured at 2 K. Purple marks are data from this work, the colored marks (blue, orange, green and red) are data from Kunakova G, et al. (2018) [26], Sondors R. et al. (2021) [36], Kunakova G, et al. (2019) [41] and Hong S. et al. (2012) [24], respectively. The light-blue shaded region is a guide to the eye (1/t), indicating a dramatic increase in sheet resistance as thickness decreases; (**c**) The three-dimensional concentration of charge carriers n_3D-Hall_ as a function of nanoribbons thickness. Orange marks are data from this work; the blue marks are data from Kunakova G, et al. (2021) [39], respectively. The light-blue shaded region is a guide to the eye, and the pink-shaded region indicates the upper bound for the bulk carrier concentration [26]; (**d**) Shubnikov–de Haas magnetoresistance oscillations as a function of 1/B for the 15 nm-thick nanoribbons measured at 2 K. The inset: the Fourier transform power spectra of ∆R_xx_(1/B) shown in (**d**); (**e**) Anti-symmetrized R_xy_(B). The blue dashed lines are the linear fit in the range of 0 to 4 T. The orange dashed lines are the linear fit in the range of 7 to 12 T. (**f**) Longitudinal (G_xx_) and transverse (G_xy_) magnetoconductance as a function of the magnetic field. The solid red lines correspond to the fit of the two-carrier model. All the data refer to the same nanoribbon as shown in (**d**).

For all measured devices, the sheet resistance linearly decreased with the decrease in temperature (a metallic transition), reaching saturation at about 30 K (Figure 6a). Such behavior was also observed previously for Bi_2_Se_3_ nanoribbons with different thicknesses [26,34] since the PVD-grown Bi_2_Se_3_ nanostructures are usually excessively doped with selenium vacancies (electron donors), which contribute to the conducting states from the bulk [2]. Such a phenomenon complicates tuning the Fermi level close/through the Dirac point via applying electrostatic gate potential to the nanostructures. Reducing the thickness of nanoribbons increases their surface-to-volume ratio, which can effectively reduce the metallic bulk conduction of Bi_2_Se_3_ nanostructures. This is evidenced by the increase in the sheet resistance of the nanoribbons with the decrease in their thickness (Figure 6b) and may help to reveal the topological surface states (TSSs) transport signatures [2].

To estimate the three-dimensional concentration of charge carriers n_3D-Hall_ of individual nanoribbons, the Hall resistance R_xy_ was measured as a function of the magnetic field at the base temperature of 2 K. The negative R_xy_(B) slope indicates n-type carriers for all measured devices (Figure 6e). The values of the carrier concentration were calculated from the Hall resistance as [39]:

$$\frac{1}{n_{3D-\mathrm{Hall}}\cdot e}=t\frac{{\mathrm{dR}}_{\mathrm{xy}}}{\mathrm{dB}}\times g,$$

where e is the elementary charge, t is the thickness of the measured nanoribbon, and g ≈ 4 (for this experiment) is a correction factor for the shunting of the Hall effect by the gold electrodes (Figure 5d) [39]. The n_3D-Hall_ values as a function of the nanoribbon thickness are presented in Figure 6c. The increase in the n_3D-Hall_ while the thickness of the nanoribbons is decreasing indicates a stronger contribution of TI surface carriers since the bulk contribution becomes less dominant [26].

To probe the TI properties of obtained nanoribbons, the magnetoresistance R_xx_ as a function of the magnetic field applied perpendicularly to the nanoribbon’s surface was measured. All devices exhibited pronounced oscillations in high magnetic fields. This effect is associated with the Shubnikov–de Haas (SdH) oscillations. After subtracting a polynomial background, the strictly periodic residual magnetoresistance ∆R_xx_ was obtained in 1/B (Figure 6d). The Fourier transform of the oscillations gives a single frequency at F = 96 T (inset of Figure 6d), which, according to the Onsager relationship, corresponds to n_2D_ = 2.32 × 10^12^ cm^−2^ [26]. Any deviation from periodicity should lead to the appearance of another (additional) frequency (corresponding to the bulk) in the Fourier transform spectrum. This was not observed, so a clear signature of a bulk-free transport nature was found in the obtained nanoribbons. Moreover, the observed SdH frequency can be attributed to the top Dirac surface states (at the interface of nanoribbon with vacuum). This is supported by our previous results [26,39], where it was shown that the charge accumulation layer formed at the nanoribbon–substrate interface dominates in the Hall conductance. Due to the overlapping of the bottom topological surface states with the accumulation layer having lower charge carrier mobility [26], the bottom surface SdH oscillations do not usually appear in the magnetoresistance. Moreover, the linear fit of Hall resistance (blue dashed line in Figure 6e) in the low magnetic field range depicts the deviation from the linearity at higher magnetic fields. This non-linearity indicates the contribution of another band (accumulation layer) with different concentrations of carriers and mobility. Indeed, the values of the 2D carrier concentrations extracted from the SdH oscillations analysis and Hall effect measurements (which include all existing types of carriers) showed a large discrepancy (n_2D SdH_ = 2.32 × 10^12^ cm^−2^ and n_2D-Hall_ = 8.93 × 10^12^ cm^−2^). Applying the two-band model analysis for the longitudinal and transverse magnetoconductance to extract the carrier concentration and the mobility of the two types of carriers [26,39], it can be assumed that one band is represented by the Dirac electrons (at the interface of the nanoribbon with vacuum) and the other band includes both the charge accumulation layer and the Dirac electrons at the bottom surface of the nanoribbon. In the fits, the value of n_1_ was set equal to n_2D-SdH_ = 2.32 × 10^12^ cm^−2^ extracted from the SdH measurements. Figure 6f shows the fitting results with the experimental data, which yields µ_1_ = 1.08 × 10^3^ cm^2^·(Vs)^−1^ for the mobility of the carriers from the topological surface states at the nanoribbon top surface, and n_2_ = 7.89 × 10^12^ cm^−2^ and µ_2_ = 5.11 × 10^2^ cm^2^·(Vs)^−1^ are representing parameters for second band. The extracted top surface mobility is in good agreement with previously published results [39,47], while the mobility of the second band is slightly lower than that published in the literature. This may be due to the possible non-uniformity of the oxide layer on the surface of the substrate, the presence of local defects, and the non-homogeneity of the accumulation layer, and may also vary from device to device.

## 4. Conclusions

A systematic study of the correlation of the main parameters (synthesis pressure and time) of the catalyst-free physical vapor deposition synthesis and the thickness of the synthesized Bi_2_Se_3_ nanoribbons showed that the optimal combination of these parameters allows the successful synthesis of ultrathin Bi_2_Se_3_ nanoribbons with thicknesses below 15 nm. Decreasing the time of heating the source material at maximal temperature during the synthesis correlated with an increased percentage of ultrathin nanoribbons, as well as a lower mean nanoribbon thickness overall. The optimal synthesis parameters for obtaining the highest yield of the Bi_2_Se_3_ nanoribbons with thicknesses below 15 nm were the temperature of the source material of 585 °C, initial pressure of the inert gas in the synthesis tube of 5 Torr, and time of heating of the source material at a maximal temperature of 0–5 min, meaning that for obtaining the ultrathin nanoribbons cooling process should start immediately or within 5 min from the moment when the maximal synthesis temperature of 585 °C has been reached.

Investigation of the transport properties of the obtained Bi_2_Se_3_ nanoribbons with thicknesses below 15 nm showed that the values of the sheet resistance for these nanoribbons are by approximately an order of magnitude higher compared to the values reported previously for the thicker Bi_2_Se_3_ nanoribbons synthesized using catalyst-free PVD. Potentially, this could lead to better tuning of the chemical potential through electrostatic gating and help access transport through topological surface states. The single frequency extracted from SdH oscillations corresponded to the topological surface states at the top surface of the nanoribbon (at the interface with the vacuum). There is no signature of 3D bulk carriers. The presence of the accumulation layer (with lower mobility) was observed at the nanoribbon–substrate interface. The topological surface states observed in the studied nanostructures confirmed that the Bi_2_Se_3_ thin (below 15 nm) nanoribbons synthesized with modified parameters do not result in a degradation of the transport properties.

## Figures and Tables

**Table 1:** Synthesis parameters, percentage of Bi_2_Se_3_ nanoribbons with thicknesses <10 nm, 10–15 nm, 15–20 nm, 20–25 nm, and >25 nm, and mean thickness (nm). In all experiments, the initial temperature T_1_ = 25 °C and heating time t_1_ = 45 min.

No.	Synthesis Parameters:					Results:					
	Temperature [°C]	Time [min]	Pressure [Torr]			Nanoribbon Thickness, nm/%					Mean Thickness [nm]
	T_m_	t_2_	p_1_	p_2_	p_3_	<10	10–15	15–20	20–25	>25	
1.	575	15	5.00	11		0%	0%	0%	0%	100%	52
2.	590	15	5.00	14		Poor quality of nanoribbons: defects in crystal structure					
3	585	30	5.00	12.8	16.2	0%	0%	2.8%	8.5%	88.7%	51
4	585	25	5.00	13.2	17	0%	0%	7.8%	7.7%	84.5%	53
5	585	20	5.00	12.4	15.2	0%	0%	20%	20%	60%	50
6	585	15	5.00	12.4	14.7	0%	2.7%	8%	16%	73.3%	36
7	585	10	5.00	13	14.8	0%	6.3%	18.8%	43.8%	31.1%	38
**8**	**585**	**5**	**5.00**	**12.3**	**13.1**	**0%**	**18.2%**	**27.3%**	**45.5%**	**9%**	**27**
**9**	**585**	**0**	**5.00**	**12.9**	**—**	**1.7%**	**13.6%**	**28.8%**	**42.4%**	**13.5%**	**24**
10	585	0	0.10	2.3	—	0%	2.7%	9.5%	27%	60.8%	53
11	585	0	1.00	4.13	—	0%	0%	10%	20%	70%	49
12	585	0	3.00	7.85	—	0%	0%	20%	20%	60%	35
13	585	0	9.00	17.9	—	0%	11.1%	11.1%	22.2%	55.6%	33
14	585	0	13.0	31.2	—	0%	0%	0%	12.5%	87.5%	30
15	585	0	18.0	40.5	—	No nanoribbons observed					

## Data Availability

The raw/processed data required to reproduce these findings cannot be shared at this time as the data also forms part of an ongoing study.
